# Association of longitudinal serum 25-hydroxyvitamin D trajectories with subsequent liver stiffness increase in patients with early-onset type 2 diabetes and metabolic dysfunction-associated steatotic liver disease

**DOI:** 10.3389/fendo.2026.1987830

**Published:** 2026-10-28

**Authors:** Hong Ji, Runzhou Pan, Xiaowei Huang

**Affiliations:** Department IV of Endocrinology and Diabetes, Cangzhou Central Hospital, Cangzhou, Hebei, China

**Keywords:** 25-hydroxyvitamin D, early-onset type 2 diabetes, liver stiffness, longitudinal trajectory, metabolic dysfunction-associated steatotic liver disease

## Abstract

**Background:**

A single serum 25-hydroxyvitamin D [25(OH)D] measurement may not adequately reflect long-term vitamin D exposure. We evaluated the associations of longitudinal 25(OH)D trajectories with subsequent liver stiffness measurement (LSM) increases in patients with early-onset type 2 diabetes and metabolic dysfunction-associated steatotic liver disease.

**Methods:**

This retrospective study included 286 patients managed between 2022 and 2025. Latent class mixed-effects modeling identified 25(OH)D trajectories before a 1-year landmark. The primary outcome was a follow-up LSM value that was both at least 20% (relative) and at least 2.0 kPa (absolute) higher than the landmark value; the conservative sensitivity outcome required increases of at least 30% and at least 3.0 kPa, respectively. Logistic regression models were fitted across 50 pseudo-class draws and pooled using Rubin’s rules. Follow-up LSM was analyzed by analysis of covariance adjusted for landmark LSM and prespecified covariates. A segmented linear mixed-effects model compared LSM slopes before and after the landmark.

**Results:**

Three trajectories were identified: persistently low (*n* = 95), increasing (*n* = 84), and stable high (*n* = 107). The primary-outcome rates were 54.7%, 33.3%, and 18.7%, respectively. Compared with the stable-high trajectory, the odds ratios in the prespecified primary model were 3.34 (95% confidence interval [CI], 1.68–6.61) for the persistently low trajectory and 1.98 (95% CI, 1.03–3.82) for the increasing trajectory. After additional adjustment for vitamin D supplementation, cumulative dose, metabolic factors, and medications, the corresponding odds ratios were 2.87 (95% CI, 1.38–5.96) and 1.84 (95% CI, 0.91–3.72). Under the conservative outcome definition, the corresponding odds ratios were 3.18 (95% CI, 1.39–7.27) and 1.71 (95% CI, 0.76–3.84). Adjusted follow-up LSM was higher by 1.07 kPa (95% CI, 0.66–1.48) and 0.52 kPa (95% CI, 0.10–0.94), respectively. The post-landmark LSM slope exceeded the pre-landmark slope by 0.98 kPa/year (95% CI, 0.80–1.16).

**Conclusions:**

The persistently low 25(OH)D trajectory showed the most consistent association with subsequent LSM increase. The increasing trajectory showed weaker, outcome-definition-dependent associations and coincided with more frequent vitamin D supplementation and a greater reduction in body mass index. These findings should be interpreted as longitudinal associations rather than causal effects or evidence of validated individual-level predictive performance.

## 1 Introduction

Early-onset type 2 diabetes is generally defined as type 2 diabetes diagnosed before 40 years of age. Its longer duration of disease exposure is associated with earlier onset of microvascular, cardiovascular, and hepatic metabolic complications (1). Metabolic dysfunction-associated steatotic liver disease (MASLD) has become a common chronic liver disease worldwide, and its burden has increased in parallel with the rising prevalence of obesity and diabetes (2). Across the natural history of MASLD, fibrosis severity is a major determinant of liver-related events and long-term prognosis (3). The new disease nomenclature emphasizes the direct link between hepatic steatosis and cardiometabolic risk factors and provides a unified framework for clinical identification and research stratification (4).

Current guidelines recommend proactive assessment of advanced liver fibrosis risk in patients with type 2 diabetes and the sequential use of blood-based scores and liver elastography (5). Clinical screening data indicate a high burden of hepatic steatosis and severe fibrosis among outpatients with type 2 diabetes, with obesity, longer disease duration, and poor metabolic control coexisting with higher liver stiffness (6). Liver surveillance in populations with diabetes remains constrained by testing resources, threshold performance, and the feasibility of repeated measurements; therefore, the information provided by dynamic biomarkers and the limits of its interpretation require clarification (7).

Serum 25-hydroxyvitamin D [25(OH)D] is the major circulating indicator of vitamin D stores and may contribute to MASLD-related pathophysiology through vitamin D receptor signaling, immune and inflammatory pathways, oxidative stress, lipid metabolism, and hepatic stellate cell activation (8). Clinical evidence based on transient elastography suggests that lower 25(OH)D levels are associated with higher liver stiffness and fibrosis indices in patients with type 2 diabetes (9). Multicenter data from China further indicate high detection rates of hepatic steatosis and liver fibrosis in patients with type 2 diabetes, with both closely related to obesity and insulin resistance (10).

A single 25(OH)D measurement is influenced by season, sunlight exposure, adiposity, diet, metabolic control, and supplement use and may not adequately characterize long-term exposure. The relationship between distinct longitudinal 25(OH)D trajectories and subsequent changes in liver stiffness measurement (LSM) has not been systematically evaluated in patients with early-onset type 2 diabetes and MASLD. Using a landmark design, this study identified 25(OH)D trajectories from baseline to 1 year and evaluated their associations with prespecified post-landmark LSM increase and follow-up LSM.

## 2 Materials and methods

### 2.1 Study population

This retrospective study was based on our hospital’s standardized integrated follow-up database for patients with early-onset type 2 diabetes and MASLD. Between January 1, 2022, and September 30, 2023, 1,026 patients entered this follow-up pathway. Of these, 702 did not have complete scheduled serial assessments spanning baseline, approximately 6 months, approximately 12 months, and approximately 24 months, whereas 324 had serial assessment records sufficient for detailed eligibility screening. Study baseline was defined as the first date between January 1, 2022, and September 30, 2023, on which serum 25(OH)D testing and liver transient elastography were performed concurrently.

Type 2 diabetes was diagnosed according to current diagnostic and classification criteria (11). Early-onset type 2 diabetes was defined as type 2 diabetes diagnosed before 40 years of age (12). MASLD was defined as hepatic steatosis with at least one cardiometabolic risk factor; all patients in this study had type 2 diabetes (13). Hepatic steatosis was confirmed by a controlled attenuation parameter (CAP) of at least 248 dB/m (14).

The inclusion criteria were as follows: (1) age of at least 18 years at study baseline; (2) clinical records sufficient to determine age at diagnosis of type 2 diabetes; (3) at least three 25(OH)D measurements between study baseline and the landmark, covering the baseline, intermediate, and pre-landmark measurement windows; (4) transient elastography data at study baseline, the landmark, and post-landmark follow-up that met prespecified quality criteria; (5) use of the same device and probe type at all time points for each patient; and (6) complete data for the primary exposure, outcomes, and prespecified core adjustment variables.

The exclusion criteria were as follows: (1) type 1 diabetes, gestational diabetes, or other specific types of diabetes; (2) mean daily ethanol intake of at least 30 g in men or at least 20 g in women, or coexisting viral hepatitis, autoimmune liver disease, drug-induced liver injury, or inherited metabolic liver disease (15); (3) cirrhosis, decompensated liver disease, or hepatic malignancy diagnosed at study baseline; (4) estimated glomerular filtration rate (eGFR) below 30 mL·min^-^¹·1.73 m^-^², dialysis, or a disorder affecting vitamin D metabolism, such as parathyroid disease or severe malabsorption; (5) use of active vitamin D analogues or vitamin D loading therapy at a dose above 50,000 international units (IU)/week for at least 4 weeks; (6) long-term use of systemic glucocorticoids or antiepileptic drugs; and (7) pregnancy or lactation.

According to Chinese adult body mass index (BMI) criteria, obesity was defined as a baseline BMI of at least 28 kg/m² (16). After application of the prespecified inclusion and exclusion criteria, 286 patients were included. This retrospective study used data collected and anonymized at our hospital. The study was reviewed and approved by the hospital ethics committee, which waived the requirement for informed consent.

The integrated follow-up pathway recommended 25(OH)D testing at baseline, approximately 6 months, and approximately 12 months, and liver transient elastography at baseline, approximately 12 months, and approximately 24 months. Scheduled examination times were determined by the follow-up calendar and were not triggered by vitamin D deficiency at baseline. Clinicians could initiate supplementation according to test results. Additional 25(OH)D tests could arise from post-supplementation safety or efficacy monitoring, routine diabetes visits, or other metabolic evaluations, but these tests did not alter the prespecified examination windows.

### 2.2 Clinical data and laboratory measurements

Two investigators trained according to a standardized protocol independently extracted data using a prespecified data dictionary. Baseline variables included age, sex, age at diabetes diagnosis, diabetes duration, smoking status, alcohol use, height, weight, waist circumference, systolic blood pressure, diastolic blood pressure, hypertension, dyslipidemia, and history of cardiovascular disease. BMI was calculated as weight in kilograms divided by height in meters squared. Change in BMI was defined as landmark BMI minus study baseline BMI; a negative value indicated a reduction in BMI. Vitamin D deficiency was defined as serum 25(OH)D below 20 ng/mL (17).

The start date, duration of continuous use, and cumulative prescribed dose of vitamin D supplements between study baseline and the landmark were recorded and converted to cholecalciferol-equivalent doses. Continuous use for at least 90 days was defined as supplementation exposure. The extended regression model used a two-part parameterization that included both an indicator of supplementation and the cumulative dose among supplemented patients; a separate descriptive stratification by supplementation status was performed within the increasing trajectory. Concurrent use of metformin, sodium-glucose cotransporter 2 (SGLT2) inhibitors, glucagon-like peptide-1 (GLP-1) receptor agonists, pioglitazone, insulin, and statins was recorded, and continuous use for at least 90 days was defined as exposure to the corresponding medication.

Laboratory variables included fasting plasma glucose, glycated hemoglobin (HbA1c), total cholesterol, triglycerides, high-density lipoprotein cholesterol, low-density lipoprotein cholesterol, alanine aminotransferase (ALT), aspartate aminotransferase (AST), γ-glutamyl transferase, albumin, platelet count, creatinine, eGFR, and uric acid. The eGFR was calculated using the 2021 Chronic Kidney Disease Epidemiology Collaboration creatinine equation without a race coefficient (18). According to the prespecified protocol, time-weighted means were calculated only for HbA1c, triglycerides, ALT, and eGFR, and at least two valid measurements between study baseline and the landmark were required for each variable. The area under the measurement-versus-time curve was calculated using the trapezoidal method and divided by the actual interval between the first and last measurements. After data entry, a second investigator verified the data, and values outside laboratory reference ranges or physiologically plausible ranges were checked against the original medical records.

### 2.3 Serum 25(OH)D measurement and identification of longitudinal trajectories

Serum 25(OH)D was measured by electrochemiluminescence on the same laboratory platform and expressed in ng/mL. The trajectory observation period extended from study baseline to the landmark. Scheduled measurement windows were study baseline, 120–240 days after baseline, and 270 days after baseline through the landmark date. The trajectory model included results that met quality criteria within these windows and additional clinically generated results outside the windows if they were obtained at least 30 days after the most recent included measurement. All measurements were modeled according to the actual blood-draw date. When multiple results were available within the same scheduled window, the result closest to the midpoint of the window was retained. Multiple measurements on the same day were averaged. For repeated measurements less than 30 days apart, only the result closest to the midpoint of the relevant scheduled window or to the most recent clinical assessment date was retained.

Longitudinal 25(OH)D trajectories were identified using latent class mixed-effects models without prespecifying the number or functional form of the trajectories. Linear and quadratic time models with two to five classes were fitted, with a subject-specific random intercept and sine and cosine terms for month of blood collection to adjust for seasonality. Each candidate model was refitted using 100 sets of distinct starting values. Model selection considered the Bayesian information criterion, Akaike information criterion, entropy, mean posterior classification probability, minimum class proportion, convergence, and clinical interpretability. Candidate models were required to converge successfully, have a minimum class proportion of at least 5%, and have a mean posterior classification probability of at least 0.70 in every class.

For descriptive analyses, trajectory classes were assigned according to maximum posterior probability. Primary logistic regression and corresponding sensitivity analyses used 50 pseudo-class draws based on each patient’s posterior class probabilities. The same model was fitted independently in each draw. The pooled coefficient was the mean of the 50 coefficient estimates; the within-draw variance (Ū) was the mean of the model-specific variance estimates; the between-draw variance (B) was the sample variance of the 50 coefficient estimates; and the total variance was calculated as T = Ū + (1 + 1/m)B, where m = 50. The pooled standard error was the square root of T and was used to calculate Wald statistics, 95% confidence intervals (CIs), and *P* values. Because 50 pseudo-class draws were used and the analysis sample was relatively large, pooled 95% CIs and *P* values were calculated using a large-sample normal approximation. Hard-classification results were reported as a separate sensitivity analysis. The baseline, 6-month, and 12-month 25(OH)D values were trajectory-defining variables and were reported descriptively without between-trajectory hypothesis testing.

To obtain a continuous patient-level measure of 25(OH)D change, a separate single-class linear mixed-effects model was fitted. The model included linear time, fixed sine and cosine effects for month of blood collection, and subject-specific random intercepts and random linear time slopes. The individual empirical Bayes slope was defined as the sum of the fixed time slope and the best linear unbiased prediction of the individual random slope and was expressed in ng/mL/year. This measure was not derived from the primary latent class model.

As a robustness analysis of the trajectory model, the three-class quadratic-time model was refitted with both a subject-specific random intercept and a random linear time slope, an unstructured intercept–slope covariance, and class-specific random-effect scales. The three-class solution was evaluated using the Akaike information criterion, Bayesian information criterion, entropy, minimum class proportion, lowest mean posterior classification probability, concordance with the primary model, and adjusted Rand index.

### 2.4 Liver transient elastography and outcome definitions

Liver transient elastography was performed after fasting for at least 3 hours, using the M or XL probe as automatically recommended by the device. The same device and probe type were used at study baseline, the landmark, and follow-up for each patient. Measurements were obtained through a right intercostal space with the patient supine and the right arm fully abducted. At least 10 valid measurements were obtained per examination. LSM was expressed as the median, and an interquartile range-to-median ratio of 0.30 or less was considered acceptable quality (19). CAP was used to assess the degree of hepatic steatosis.

An LSM examination performed 330–400 days after study baseline was defined as the landmark examination, and an examination performed 330–400 days after the landmark was defined as the follow-up examination. If acute liver injury, biliary obstruction, hepatic congestion, or an ALT level more than five times the upper limit of normal was present at the time of examination, that LSM result was not used for outcome ascertainment; instead, the nearest quality-assured result obtained during clinical stability within the prespecified window was selected.

The primary outcome was a prespecified post-landmark LSM increase, defined as a follow-up LSM value that was both at least 20% and at least 2.0 kPa higher than the landmark LSM value. The conservative sensitivity outcome required both a relative increase of at least 30% and an absolute increase of at least 3.0 kPa to reduce the influence of short-term measurement variability in the lower LSM range. The secondary continuous outcome was follow-up LSM, analyzed by analysis of covariance with landmark LSM included as the baseline covariate. The alternative outcome was an increase of at least one LSM risk category, with prespecified categories of <8.0, 8.0 to <12.0, and ≥12.0 kPa (20). Two investigators who were unaware of the 25(OH)D trajectory class independently adjudicated the binary outcomes; disagreements were reviewed by a third investigator.

### 2.5 Statistical analysis

Statistical analyses were performed using R software. The distributions of continuous variables were assessed using histograms, quantile–quantile (Q–Q) plots, and the Shapiro–Wilk test. Approximately normally distributed variables are presented as mean ± standard deviation and were compared across the three groups using one-way analysis of variance. Skewed variables are presented as median [interquartile range] and were compared using the Kruskal–Wallis test. Categorical variables are presented as *n* (%) and were compared using the Pearson *χ²* test, with Fisher exact test used when the expected frequency in any cell was below 5.

Demographic and clinical characteristics, medication data, and laboratory variables were treated as three separate exploratory testing families. The Benjamini–Hochberg method was used to control the false discovery rate, and both raw *P* values and adjusted *q* values were reported; *q* < 0.05 within the corresponding testing family was considered statistically significant. No between-group hypothesis testing was performed for trajectory-defining variables.

BMI was analyzed using a linear mixed-effects model that included trajectory class, time, and a trajectory class × time interaction, with a subject-specific random intercept. CAP was analyzed using the same model across study baseline, the landmark, and follow-up. Degrees of freedom for fixed-effect tests were approximated using the Satterthwaite method.

Logistic regression was used to evaluate the association between 25(OH)D trajectories and the prespecified LSM increase. Model 1 included trajectory class only. Model 2 was the prespecified primary adjusted model and included trajectory class, age, sex, diabetes duration, baseline BMI, change in BMI, time-weighted HbA1c, baseline CAP, and study-baseline LSM. Model 3 was the extended adjusted model and additionally included time-weighted triglycerides, time-weighted ALT, time-weighted eGFR, vitamin D supplementation, cumulative dose among supplemented patients, metformin, SGLT2 inhibitors, GLP-1 receptor agonists, pioglitazone, insulin, and statins. Because vitamin D supplementation occurred during trajectory formation and may have been influenced by prior 25(OH)D levels, adjustment for supplementation was prespecified in the extended model, and treatment-related confounding was further evaluated in an analysis excluding supplemented patients. Model 3 used Firth penalized maximum likelihood estimation to reduce small-sample bias in the multiparameter model. Odds ratios (ORs), 95% CIs, Wald *z* statistics, and *P* values were reported. The overall effect of trajectory class was assessed using a joint Wald *χ²* test. A variance inflation factor above 5 was considered indicative of potentially important multicollinearity.

To distinguish the initial 25(OH)D concentration from longitudinal change, trajectory class in Model 2 was jointly replaced by baseline 25(OH)D and the individual empirical Bayes slope from the separate random-intercept plus random-slope model. The time-weighted mean 25(OH)D concentration was also evaluated separately. Three-knot restricted cubic splines were used to test for nonlinear associations of baseline BMI, time-weighted HbA1c, baseline CAP, baseline LSM, baseline 25(OH)D, and the 25(OH)D change slope with the outcome.

The continuous post-landmark LSM outcome was analyzed by analysis of covariance, with follow-up LSM as the dependent variable and trajectory class as the primary independent variable. The model adjusted for landmark LSM, the actual interval from landmark to follow-up, age, sex, diabetes duration, baseline BMI, change in BMI, time-weighted HbA1c, time-weighted triglycerides, time-weighted ALT, time-weighted eGFR, baseline CAP, the two-part vitamin D supplementation variables, and medication exposures. Adjusted marginal means for each trajectory group and adjusted mean differences relative to the stable-high group were reported. A trajectory class × landmark LSM interaction was used to test homogeneity of regression slopes, and heteroskedasticity-consistent type 3 (HC3) robust standard errors were used to evaluate the influence of heteroskedasticity. The primary analysis of covariance used maximum-posterior-probability classification and was repeated across 50 pseudo-class draws to evaluate classification uncertainty. A linear mixed-effects model with a subject-specific random intercept was used as a supporting analysis.

To compare temporal trends across the two LSM observation phases, a segmented linear mixed-effects model with a knot at the landmark was fitted. Time was calculated in years from the actual examination dates. The model included phase-specific time terms and a subject-specific random intercept and adjusted for age, sex, diabetes duration, baseline BMI, change in BMI, time-weighted HbA1c, and baseline CAP. A linear contrast was used to test the difference between the landmark-to-follow-up slope and the baseline-to-landmark slope. The analysis was repeated after excluding patients in the highest 2.5% of absolute LSM change from the landmark to follow-up.

Sensitivity analyses included (1) maximum-posterior-probability hard classification; (2) exclusion of patients receiving vitamin D supplementation; (3) an increase in LSM risk category as the alternative outcome; (4) the conservative outcome requiring a relative increase of at least 30% and an absolute increase of at least 3.0 kPa; (5) restriction to patients with a posterior classification probability of at least 0.80; (6) omission of study-baseline LSM; (7) additional inclusion of landmark LSM in the primary adjusted model; and (8) follow-up LSM as a continuous outcome, adjusted for landmark LSM and prespecified covariates. Each binary sensitivity analysis used the covariate set from Model 2. The conservative outcome was analyzed using Firth penalized logistic regression, whereas the remaining binary sensitivity analyses used standard logistic regression.

Within the increasing trajectory, patients were descriptively stratified according to whether they had received continuous vitamin D supplementation for at least 90 days before the landmark. Baseline, 6-month, and 12-month 25(OH)D levels and the change from baseline to 12 months were reported. Between-group comparisons at the three time points used Welch *t* tests with Holm adjustment, and change scores were compared using a Welch *t* test.

Effect modification by sex and baseline obesity status was prespecified. Interactions were evaluated in the full sample using Firth penalized logistic regression models that included trajectory class, the main effect of the stratification variable, and their interaction. The sex-interaction model adjusted for all covariates in the primary model except sex. The obesity-interaction model replaced continuous baseline BMI with the binary obesity indicator and retained the remaining covariates. The two interaction tests were adjusted using the Holm method.

Descriptive analyses used available cases, and the corresponding valid sample sizes are reported in the tables. Data were complete for the primary exposure, outcomes, and covariates required for the primary model; therefore, clinical variables were not imputed. All tests were two-sided, and *P* < 0.05 was considered statistically significant.

## 3 Results

### 3.1 Study population and longitudinal 25(OH)D trajectories

Among the 1,026 patients who entered the integrated follow-up pathway, 324 had the scheduled serial assessment data required for detailed eligibility screening. Thirty-eight patients were excluded: 12 because the number or timing of 25(OH)D measurements did not meet the requirements; 8 because liver transient elastography data were missing or failed to meet quality criteria; 4 because the device or probe type differed across time points; 6 because of other causes of liver disease; 3 because cirrhosis or hepatic malignancy was present at baseline; 3 because of severe renal dysfunction or a disorder affecting vitamin D metabolism; 1 because of vitamin D loading therapy; and 1 because of pregnancy or lactation. The final analysis included 286 patients.

At baseline, 173 patients had 25(OH)D levels below 20 ng/mL and 113 had levels of at least 20 ng/mL. The median number of 25(OH)D measurements during the trajectory observation period was 4 (3, 4) in both groups (*P* = 0.554), and the median interval from baseline to the first repeat measurement was 178[162, 196] days and 180[164, 199] days, respectively (*P* = 0.517). In addition to the three scheduled measurement times, 154 patients (53.8%) had at least one eligible additional test, for a total of 164 additional tests. Of these, 92 (56.1%) were performed for monitoring after vitamin D supplementation, 49 (29.9%) during routine diabetes visits, and 23 (14.0%) during other metabolic evaluations. Additional testing occurred in 55.5% (96/173) of patients with baseline deficiency and 51.3% (58/113) of those without baseline deficiency, with no statistically significant difference (*χ²* = 0.477, *P* = 0.490).

The mean age was 38.1 ± 5.7 years, and 164 patients (57.3%) were men. The mean age at diabetes diagnosis was 30.8 ± 4.2 years, and the median diabetes duration was 6.4 [4.2, 9.4] years. A total of 1,022 valid 25(OH)D measurements were obtained during the trajectory observation period, with a median of 4 (3, 4) measurements per patient. A total of 858 quality-assured liver transient elastography examinations were included. The median number of valid measurements per examination was 11 (10, 12), and the median interquartile range-to-median ratio was 0.18 [0.14, 0.22]. The M probe was used in 231 patients and the XL probe in 55 patients, with no change in probe type during follow-up.

All eight candidate trajectory models converged successfully. Among models meeting the prespecified criteria for class proportion and posterior classification quality, the three-class quadratic time random-intercept model had the lowest Bayesian information criterion; the minimum class proportions in the four-class and five-class quadratic time models were below 5% (**Supplementary Table S1**). The three-class quadratic time model with both random intercepts and random slopes also identified persistently low, increasing, and stable-high patterns. Classification agreed with the primary model in 262 patients (91.6%), and the adjusted Rand index was 0.81. On the basis of the lower Bayesian information criterion and greater parsimony, the random-intercept model was retained as the primary trajectory model (**Supplementary Table S2**).

Using maximum-posterior-probability classification, 95 patients (33.2%) were assigned to the persistently low trajectory, 84 (29.4%) to the increasing trajectory, and 107 (37.4%) to the stable-high trajectory. The corresponding mean posterior classification probabilities were 0.89, 0.87, and 0.91, respectively. Posterior classification probabilities were at least 0.70 in 281 patients (98.3%) and at least 0.80 in 267 patients (93.4%). The persistently low trajectory remained low throughout the observation period, the increasing trajectory rose over time, and the stable-high trajectory remained high and stable (**Figure 1**).

**Figure 1:**
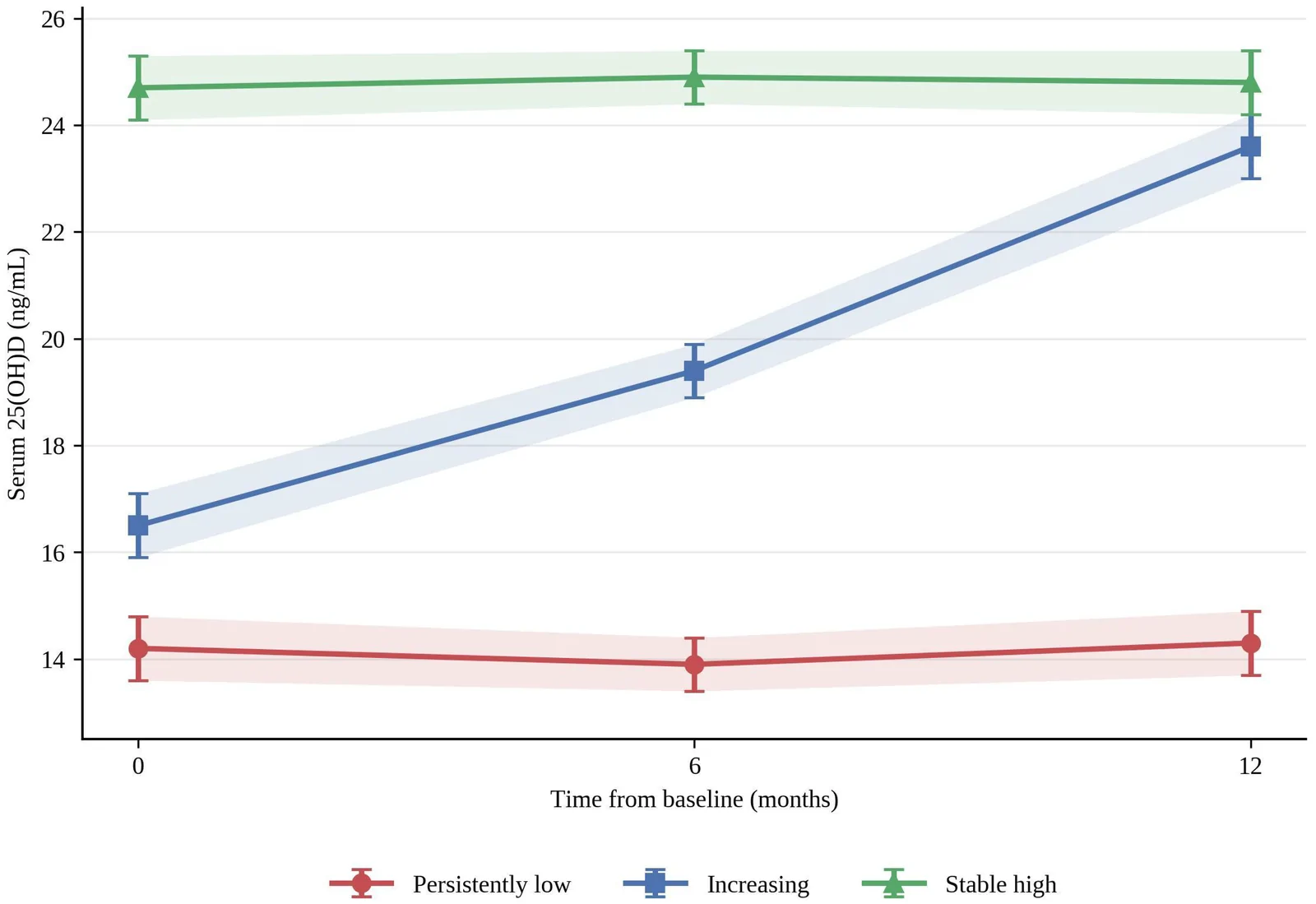
Longitudinal trajectories of serum 25-hydroxyvitamin D [25(OH)D]. The x-axis represents time from study baseline (months), and the y-axis represents the 25(OH)D concentration (ng/mL) estimated using a three-class quadratic-time latent class mixed-effects model. The persistently low, increasing, and stable-high trajectories included 95, 84, and 107 patients, respectively. Points indicate model-estimated means at baseline, 6 months, and 12 months; error bars and shaded areas indicate 95% confidence intervals (CIs). The model included a subject-specific random intercept and sine and cosine terms for month of blood collection. Because 25(OH)D values were used to construct the trajectories, between-trajectory hypothesis tests are not reported.

### 3.2 Demographic, clinical, medication, and laboratory characteristics

Among demographic and clinical characteristics, weight and BMI decreased sequentially from the persistently low group to the increasing group and the stable-high group, with statistically significant differences after false-discovery-rate adjustment. Age, sex, age at diabetes diagnosis, diabetes duration, height, waist circumference, systolic blood pressure, diastolic blood pressure, smoking, alcohol use, hypertension, dyslipidemia, history of cardiovascular disease, and probe type did not differ significantly after adjustment (all *q* > 0.05). Among medication exposures, the proportion receiving vitamin D supplementation and the cumulative dose among supplemented patients were highest in the increasing group, with statistically significant differences. Time to first supplementation and use of metformin, SGLT2 inhibitors, GLP-1 receptor agonists, pioglitazone, insulin, and statins did not differ significantly after false-discovery-rate adjustment (all *q* > 0.05). Among laboratory variables, fasting plasma glucose, HbA1c, triglycerides, low-density lipoprotein cholesterol, creatinine, uric acid, time-weighted HbA1c, time-weighted triglycerides, and time-weighted ALT were generally highest in the persistently low group and lowest in the stable-high group. AST was higher in the increasing group, albumin was lower in the persistently low group, and platelet count was lower in the increasing group; these differences were statistically significant. Total cholesterol, high-density lipoprotein cholesterol, ALT, γ-glutamyl transferase, eGFR, and time-weighted eGFR did not differ significantly (all *q* > 0.05) (**Table 1**).

**Table 1:** Demographic, clinical, medication, and laboratory characteristics across 25(OH)D trajectory groups.

Variable	Persistently low (*n* = 95)	Increasing (*n* = 84)	Stable high (*n* = 107)	Statistic	*P* value	*q* value
Demographic and clinical characteristics						
Age, years	37.8 ± 5.7	38.6 ± 6.1	38.1 ± 5.3	*F* = 0.449	0.639	0.897
Men, *n* (%)	57 (60.0)	52 (61.9)	55 (51.4)	*χ²* = 2.533	0.282	0.631
Age at diabetes diagnosis, years	30.3 ± 4.1	30.9 ± 3.9	31.3 ± 4.4	*F* = 1.463	0.233	0.622
Diabetes duration, years	6.9 [4.5, 9.2]	6.4 [4.3, 9.6]	6.1 [3.8, 9.1]	*H* = 2.306	0.316	0.631
Height, cm	167.7 ± 7.1	167.3 ± 7.1	166.9 ± 7.6	*F* = 0.303	0.739	0.897
Weight, kg	84.0 ± 11.7	80.2 ± 10.7	78.3 ± 12.4	*F* = 6.115	0.003	0.020
Waist circumference, cm	96.2 ± 5.5 (*n* = 89)	95.2 ± 6.0 (*n* = 78)	95.1 ± 5.6 (*n* = 101)	*F* = 1.033	0.357	0.635
BMI, kg/m²	29.8 ± 3.1	28.6 ± 3.4	28.0 ± 3.7	*F* = 7.116	0.001	0.015
Systolic blood pressure, mmHg	129.3 ± 13.3	130.2 ± 13.7	130.6 ± 13.4	*F* = 0.242	0.785	0.897
Diastolic blood pressure, mmHg	82.2 ± 8.5	82.1 ± 7.8	80.9 ± 8.3	*F* = 0.781	0.459	0.735
Current smoking, *n* (%)	31 (32.6)	26 (31.0)	21 (19.6)	*χ²* = 5.103	0.078	0.249
Current alcohol use, *n* (%)	17 (17.9)	16 (19.0)	17 (15.9)	*χ²* = 0.342	0.843	0.899
Hypertension, *n* (%)	51 (53.7)	29 (34.5)	45 (42.1)	*χ²* = 6.841	0.033	0.174
Dyslipidemia, *n* (%)	66 (69.5)	54 (64.3)	58 (54.2)	*χ²* = 5.204	0.074	0.249
History of cardiovascular disease, *n* (%)	7 (7.4)	4 (4.8)	8 (7.5)	*χ²* = 0.680	0.712	0.897
M probe, *n* (%)	77 (81.1)	67 (79.8)	87 (81.3)	*χ²* = 0.080	0.961	0.961
Medication exposure before the landmark						
Vitamin D supplementation, *n* (%)	16 (16.8)	50 (59.5)	25 (23.4)	*χ²* = 43.068	<0.001	<0.001
Metformin, *n* (%)	74 (77.9)	71 (84.5)	86 (80.4)	*χ²* = 1.278	0.528	0.528
SGLT2 inhibitors, *n* (%)	35 (36.8)	39 (46.4)	37 (34.6)	*χ²* = 3.014	0.222	0.249
GLP-1 receptor agonists, *n* (%)	17 (17.9)	24 (28.6)	17 (15.9)	*χ²* = 5.183	0.075	0.102
Pioglitazone, *n* (%)	10 (10.5)	19 (22.6)	21 (19.6)	*χ²* = 5.064	0.080	0.102
Insulin, *n* (%)	35 (36.8)	28 (33.3)	21 (19.6)	*χ²* = 8.090	0.018	0.053
Statins, *n* (%)	56 (58.9)	43 (51.2)	44 (41.1)	*χ²* = 6.464	0.039	0.089
Cumulative dose among supplemented patients, ×10^4^ IU	12 [9, 18] (*n* = 16)	18 [12, 24] (*n* = 50)	12 [9, 18] (*n* = 25)	*H* = 12.351	0.002	0.009
Time from baseline to first supplementation, days	93 [45, 168]	58 [22, 105]	86 [38, 149]	*H* = 5.626	0.060	0.102
Laboratory variables						
Fasting plasma glucose, mmol/L	8.8 [7.9, 10.4]	7.9 [6.9, 9.7]	8.0 [6.6, 9.4]	*H* = 15.573	<0.001	0.002
HbA1c, %	8.29 ± 1.06	7.96 ± 0.92	7.77 ± 1.11	*F* = 6.364	0.002	0.006
Total cholesterol, mmol/L	4.89 ± 0.87	4.82 ± 0.98	4.71 ± 0.92	*F* = 0.981	0.376	0.398
Triglycerides, mmol/L	2.17 [1.64, 2.46]	1.74 [1.34, 2.25]	1.60 [1.26, 2.07]	*H* = 24.500	<0.001	<0.001
High-density lipoprotein cholesterol, mmol/L	1.04 ± 0.21	1.08 ± 0.21	1.10 ± 0.22	*F* = 2.025	0.134	0.172
Low-density lipoprotein cholesterol, mmol/L	2.96 ± 0.68	2.87 ± 0.66	2.69 ± 0.76	*F* = 3.853	0.022	0.037
ALT, U/L	39.8 [29.3, 53.2]	39.7 [29.4, 53.9]	35.2 [27.2, 45.0]	*H* = 5.000	0.082	0.114
AST, U/L	28.4 [23.3, 36.4]	28.7 [24.3, 35.2]	25.9 [20.5, 31.8]	*H* = 7.054	0.029	0.044
γ-Glutamyl transferase, U/L	45.6 [31.7, 69.1]	42.7 [31.2, 64.9]	38.5 [28.5, 58.2]	*H* = 3.621	0.164	0.196
Albumin, g/L	42.3 ± 2.4	43.1 ± 2.7	43.4 ± 2.2	*F* = 5.431	0.005	0.011
Platelet count, ×10^9^/L	233.4 ± 46.9	223.2 ± 49.2	247.4 ± 46.0	*F* = 6.331	0.002	0.006
Creatinine, μmol/L	77.5 [65.0, 85.0]	70.5 [63.8, 80.4]	68.5 [58.3, 77.6]	*H* = 11.696	0.003	0.007
eGFR, mL·min^-^¹·1.73 m^-^²	111.2 ± 9.5	111.8 ± 9.5	112.1 ± 10.0	*F* = 0.222	0.801	0.801
Uric acid, μmol/L	374.4 ± 67.3 (*n* = 91)	362.9 ± 69.2 (*n* = 80)	346.2 ± 72.2 (*n* = 101)	*F* = 3.981	0.020	0.036
Time-weighted HbA1c, %	8.24 ± 1.12	7.70 ± 0.99	7.68 ± 1.16	*F* = 7.986	<0.001	0.002
Time-weighted triglycerides, mmol/L	2.13 [1.64, 2.60]	1.60 [1.27, 2.20]	1.57 [1.19, 2.12]	*H* = 26.771	<0.001	<0.001
Time-weighted ALT, U/L	42.1 [29.7, 53.9]	35.5 [26.3, 50.5]	34.0 [24.9, 43.1]	*H* = 9.310	0.010	0.019
Time-weighted eGFR, mL·min^-^¹·1.73 m^-^²	109.6 ± 10.1	111.5 ± 10.5	111.7 ± 10.6	*F* = 1.194	0.304	0.342

Data are presented as mean ± standard deviation, median [interquartile range], or *n* (%). Mean ± standard deviation is used for approximately normally distributed continuous variables, median [interquartile range] for skewed continuous variables, and *n* (%) for categorical variables. *F* is the one-way analysis-of-variance statistic, *H* is the Kruskal–Wallis statistic, and *χ²* is the Pearson chi-square statistic. *P* values are raw two-sided probabilities; *q* values are *P* values adjusted using the Benjamini–Hochberg method to control the false discovery rate within each prespecified testing family. Demographic and clinical characteristics, medication exposures, and laboratory variables constituted three separate exploratory testing families. The valid sample size was 268 for waist circumference and 272 for uric acid. 25(OH)D, 25-hydroxyvitamin D; BMI, body mass index; SGLT2, sodium-glucose cotransporter 2; GLP-1, glucagon-like peptide-1; HbA1c, glycated hemoglobin; ALT, alanine aminotransferase; AST, aspartate aminotransferase; eGFR, estimated glomerular filtration rate; IU, international units.

### 3.3 Stratification by vitamin D supplementation within the increasing trajectory

Within the increasing trajectory, 50 patients (59.5%) received vitamin D supplementation and 34 (40.5%) did not. Baseline 25(OH)D levels were similar between the two subgroups (*P* = 0.285), and levels increased over time in both. Supplemented patients had higher levels at 6 months and 12 months and a greater increase from baseline to 12 months (Holm-adjusted *P* = 0.004, *P* < 0.001, and *P* < 0.001, respectively) (**Supplementary Table S3**).

### 3.4 Longitudinal measures and LSM outcomes

The persistently low trajectory remained low at all three time points, the increasing trajectory rose over time, and the stable-high trajectory remained high and stable. The number of measurements did not differ significantly among the three groups, and the distribution of baseline deficiency was reported descriptively as a trajectory-defining feature. Baseline and landmark BMI were higher in the persistently low group, whereas the increasing group had the greatest reduction in BMI; the trajectory class × time interaction for BMI was statistically significant. CAP remained higher in the persistently low group and decreased in the other two groups, with a statistically significant CAP interaction. Baseline LSM did not differ significantly among groups. Landmark LSM, follow-up LSM, and annualized change from landmark to follow-up decreased sequentially from the persistently low group to the stable-high group, whereas annualized change from baseline to landmark and the landmark-to-follow-up interval did not differ significantly. The proportions with prespecified LSM increase, the conservative LSM increase outcome, and an increase in LSM risk category were highest in the persistently low group and lowest in the stable-high group (all *P* < 0.01) (**Table 2**).

**Table 2:** Longitudinal measures and LSM outcomes across 25(OH)D trajectory groups.

Variable	Persistently low (*n* = 95)	Increasing (*n* = 84)	Stable high (*n* = 107)	Statistic	*P* value
Baseline 25(OH)D, ng/mL	14.1 ± 3.6	16.6 ± 2.9	24.6 ± 3.6	—†	—†
6-month 25(OH)D, ng/mL	13.9 ± 2.5	19.3 ± 2.8	24.9 ± 3.5	—†	—†
12-month 25(OH)D, ng/mL	14.4 ± 2.9	23.5 ± 2.5	24.8 ± 3.4	—†	—†
Number of 25(OH)D measurements	3 [3, 4]	4 [3, 4]	4 [3, 4]	*H* = 4.724	0.094
Baseline 25(OH)D <20 ng/mL, *n* (%)	87 (91.6)	74 (88.1)	12 (11.2)	—†	—†
Baseline BMI, kg/m²	29.80 ± 3.10	28.60 ± 3.40	28.00 ± 3.70	—‡	—‡
Landmark BMI, kg/m²	29.76 ± 3.16	27.77 ± 3.53	27.71 ± 3.78	—‡	—‡
Change in BMI, kg/m²	−0.04 ± 1.02	−0.83 ± 0.97	−0.29 ± 0.94	—‡	—‡
BMI trajectory class × time	—	—	—	*F* = 15.118	<0.001
Baseline CAP, dB/m	311.8 ± 23.4	300.5 ± 21.8	294.9 ± 25.0	—‡	—‡
Landmark CAP, dB/m	311.9 ± 27.2	293.1 ± 23.3	290.3 ± 28.6	—‡	—‡
Follow-up CAP, dB/m	313.9 ± 29.5	290.1 ± 23.9	289.0 ± 30.1	—‡	—‡
CAP trajectory class × time	—	—	—	*F* = 9.842	<0.001
Baseline LSM, kPa	7.28 [6.18, 8.15]	6.54 [5.78, 7.58]	6.82 [5.93, 7.98]	*H* = 5.282	0.071
Landmark LSM, kPa	7.55 [6.15, 8.56]	6.69 [5.86, 7.81]	6.64 [5.95, 7.81]	*H* = 7.427	0.024
Annualized LSM change from baseline to landmark, kPa/year	0.18 [−0.31, 0.66]	0.11 [−0.35, 0.58]	−0.06 [−0.49, 0.42]	*H* = 4.012	0.135
Follow-up LSM, kPa	9.14 [7.61, 10.98]	7.90 [6.62, 9.35]	7.07 [5.95, 8.72]	*H* = 26.637	<0.001
Annualized LSM change from landmark to follow-up, kPa/year	2.30 [0.67, 2.85]	0.77 [0.32, 2.38]	0.19 [−0.15, 0.67]	*H* = 48.287	<0.001
Landmark-to-follow-up interval, days	366 [354, 378]	367 [356, 380]	365 [354, 377]	*H* = 1.126	0.570
Prespecified LSM increase, *n* (%)	52 (54.7)	28 (33.3)	20 (18.7)	*χ²* = 28.891	<0.001
Conservative LSM increase outcome, *n* (%)	31 (32.6)	15 (17.9)	9 (8.4)	*χ²* = 19.150	<0.001
Increase in LSM risk category by ≥1 level, *n* (%)	38 (40.0)	27 (32.1)	20 (18.7)	*χ²* = 11.273	0.004

Data are presented as mean ± standard deviation, median [interquartile range], or *n* (%). *F* is the *F* statistic for the trajectory class × time interaction in the linear mixed-effects model, *H* is the Kruskal–Wallis statistic, and *χ²* is the Pearson chi-square statistic; *P* values are two-sided probabilities. ^†^Trajectory-defining variables are reported descriptively without between-trajectory hypothesis testing. ^‡^Raw repeated-measurement values; longitudinal between-group differences were evaluated using the trajectory class × time interaction. The conservative LSM increase outcome was defined as a follow-up LSM that was both at least 30% and at least 3.0 kPa higher than landmark LSM. 25(OH)D, 25-hydroxyvitamin D; BMI, body mass index; CAP, controlled attenuation parameter; LSM, liver stiffness measurement.

The two investigators agreed on the initial classification of the primary outcome in 279 of 286 patients (97.6%); Cohen’s *κ* was 0.946 (95% CI, 0.907–0.986). The seven remaining disagreements were adjudicated by the third investigator.

### 3.5 Association of 25(OH)D trajectories with prespecified LSM increase

In the unadjusted model, the odds of the prespecified LSM increase were higher for both the increasing and persistently low trajectories than for the stable-high trajectory (*P* = 0.017 and *P* < 0.001, respectively). In the primary adjusted model, the odds remained higher for both trajectories (*P* = 0.041 and *P* < 0.001, respectively). In the extended Firth model, the persistently low trajectory remained statistically associated with the outcome (*P* = 0.005), whereas the increasing trajectory was not statistically significant (*P* = 0.091). The overall effect of trajectory class was statistically significant in all three models (all *P* < 0.01) (**Table 3**).

**Table 3:** Association of 25(OH)D trajectories with prespecified LSM increase across sequentially adjusted models.

Model	Trajectory class	*β*	SE	Wald *z*	OR (95% CI)	*P* value
Model 1	Increasing	0.760	0.319	2.382	2.14 (1.14–4.00)	0.017
Model 1	Persistently low	1.622	0.333	4.871	5.06 (2.64–9.72)	<0.001
Model 2: primary adjusted model	Increasing	0.684	0.335	2.042	1.98 (1.03–3.82)	0.041
Model 2: primary adjusted model	Persistently low	1.205	0.349	3.453	3.34 (1.68–6.61)	<0.001
Model 3: extended Firth model	Increasing	0.609	0.360	1.692	1.84 (0.91–3.72)	0.091
Model 3: extended Firth model	Persistently low	1.054	0.373	2.826	2.87 (1.38–5.96)	0.005

The stable-high trajectory was the reference. Model 1 included trajectory class only. Model 2 adjusted for age, sex, diabetes duration, baseline BMI, change in BMI, time-weighted HbA1c, baseline CAP, and study-baseline LSM. Model 3 used Firth penalized maximum-likelihood logistic regression and additionally adjusted for time-weighted triglycerides, time-weighted ALT, time-weighted eGFR, the two-part vitamin D supplementation variables, and medication exposures. Results were based on 50 pseudo-class draws, with within-draw and between-draw variances combined using Rubin’s rules. *β* denotes the regression coefficient for the log odds, SE denotes the Rubin-pooled standard error, Wald *z* is *β*/SE, OR is exp(*β*), 95% CIs were derived from *β* ± 1.96 × SE, and *P* denotes the two-sided Wald-test probability based on a large-sample normal approximation. 25(OH)D, 25-hydroxyvitamin D; BMI, body mass index; HbA1c, glycated hemoglobin; CAP, controlled attenuation parameter; LSM, liver stiffness measurement; ALT, alanine aminotransferase; eGFR, estimated glomerular filtration rate; OR, odds ratio; CI, confidence interval.

In the extended Firth model, the persistently low trajectory, time-weighted HbA1c, baseline CAP, and study-baseline LSM remained statistically associated with the prespecified LSM increase. The 95% CIs for the increasing trajectory and baseline BMI included 1. Age, sex, diabetes duration, change in BMI, time-weighted triglycerides, time-weighted ALT, time-weighted eGFR, vitamin D supplementation, cumulative dose among supplemented patients, metformin, SGLT2 inhibitors, GLP-1 receptor agonists, pioglitazone, insulin, and statins were not statistically significant (all *P* > 0.05) (**Table 4**).

**Table 4:** Extended firth-adjusted model for prespecified LSM increase.

Variable	*β*	SE	Wald *z*	OR (95% CI)	*P* value
Increasing vs stable-high trajectory	0.609	0.360	1.692	1.84 (0.91–3.72)	0.091
Persistently low vs stable-high trajectory	1.054	0.373	2.826	2.87 (1.38–5.96)	0.005
Age, per 5-year increase	0.030	0.121	0.248	1.03 (0.81–1.31)	0.804
Men vs women	−0.073	0.239	−0.305	0.93 (0.58–1.49)	0.760
Diabetes duration, per 3-year increase	0.095	0.101	0.941	1.10 (0.90–1.34)	0.347
Baseline BMI, per 1 kg/m² increase	0.077	0.041	1.878	1.08 (1.00–1.17)	0.060
Change in BMI, per 1 kg/m² increase	0.131	0.110	1.191	1.14 (0.92–1.41)	0.234
Time-weighted HbA1c, per 1% increase	0.300	0.121	2.479	1.35 (1.06–1.71)	0.013
Time-weighted triglycerides, per 1 mmol/L increase	0.095	0.091	1.044	1.10 (0.92–1.31)	0.297
Time-weighted ALT, per 10 U/L increase	0.058	0.058	1.000	1.06 (0.95–1.19)	0.317
Time-weighted eGFR, per 10 mL·min^-^¹·1.73 m^-^² increase	−0.030	0.089	−0.337	0.97 (0.82–1.16)	0.736
Baseline CAP, per 10 dB/m increase	0.104	0.045	2.311	1.11 (1.02–1.21)	0.021
Study-baseline LSM, per 1 kPa increase	0.174	0.067	2.597	1.19 (1.04–1.36)	0.009
Vitamin D supplementation, yes vs no	−0.041	0.337	−0.122	0.96 (0.50–1.86)	0.903
Cumulative dose among supplemented patients, per 100,000 IU increase	−0.083	0.117	−0.709	0.92 (0.73–1.16)	0.478
Metformin, yes vs no	−0.128	0.347	−0.369	0.88 (0.45–1.74)	0.712
SGLT2 inhibitors, yes vs no	−0.198	0.278	−0.712	0.82 (0.48–1.41)	0.476
GLP-1 receptor agonists, yes vs no	−0.223	0.336	−0.664	0.80 (0.41–1.55)	0.507
Pioglitazone, yes vs no	−0.094	0.344	−0.273	0.91 (0.46–1.79)	0.785
Insulin, yes vs no	0.182	0.289	0.630	1.20 (0.68–2.11)	0.529
Statins, yes vs no	0.077	0.274	0.281	1.08 (0.63–1.85)	0.779

The model used Firth penalized maximum-likelihood logistic regression. It was refitted separately in each of 50 pseudo-class draws, and all regression coefficients and covariance matrices were pooled using Rubin’s rules to combine within-draw and between-draw variances. Pooled 95% CIs and *P* values were calculated using a large-sample normal approximation. Landmark LSM was not included in the primary extended model; results after additional adjustment for this variable are reported separately in the sensitivity analyses. *β* denotes the regression coefficient for the log odds, SE denotes the Rubin-pooled standard error, Wald *z* is *β*/SE, OR is exp(*β*), 95% CIs were derived from *β* ± 1.96 × SE, and *P* denotes the two-sided Wald-test probability. BMI, body mass index; HbA1c, glycated hemoglobin; ALT, alanine aminotransferase; eGFR, estimated glomerular filtration rate; CAP, controlled attenuation parameter; LSM, liver stiffness measurement; SGLT2, sodium-glucose cotransporter 2; GLP-1, glucagon-like peptide-1; IU, international units; OR, odds ratio; CI, confidence interval.

### 3.6 Joint analysis of baseline 25(OH)D and longitudinal change

After trajectory class was jointly replaced by baseline 25(OH)D and the individual empirical Bayes slope from the separate random-intercept plus random-slope model, the adjusted OR was 0.86 (95% CI, 0.71–1.04; *P* = 0.121) for each 5-ng/mL increase in baseline 25(OH)D and 0.74 (95% CI, 0.58–0.94; *P* = 0.014) for each 5-ng/mL/year increase in the annual change slope. When time-weighted mean 25(OH)D was analyzed separately, the adjusted OR was 0.80 (95% CI, 0.68–0.94; *P* = 0.007) per 5-ng/mL increase.

Restricted cubic spline analyses provided no evidence of nonlinear associations between the prespecified LSM increase and baseline BMI, time-weighted HbA1c, baseline CAP, baseline LSM, baseline 25(OH)D, or the 25(OH)D change slope; the corresponding *P* values for nonlinearity were 0.421, 0.310, 0.583, 0.270, 0.285, and 0.415, respectively.

### 3.7 Continuous post-landmark LSM outcome

Trajectory class was associated with follow-up LSM overall (*F* = 13.760; degrees of freedom, 2 and 263; *P* < 0.001). After adjustment for landmark LSM, the actual follow-up interval, and prespecified clinical, metabolic, and medication variables, follow-up LSM increased sequentially from the stable-high group to the increasing group and the persistently low group. Both the increasing and persistently low groups had higher follow-up LSM than the stable-high group (*P* = 0.016 and *P* < 0.001, respectively) (**Table 5**; **Figure 2**). The trajectory class × landmark LSM interaction was not statistically significant (*F* = 1.120; degrees of freedom, 2 and 261; *P* = 0.328). HC3 robust standard errors and the sensitivity analysis across 50 pseudo-class draws yielded between-group differences in the same direction. In the supporting repeated-measures model, the subject-specific random-intercept variance was 1.58 kPa² and the residual variance was 0.82 kPa²; the model-estimated time trends were 0.43, 0.81, and 1.55 kPa/year in the stable-high, increasing, and persistently low groups, respectively.

**Table 5:** Adjusted follow-up LSM across 25(OH)D trajectory groups.

Trajectory group	Adjusted follow-up LSM, kPa (95% CI)	Adjusted mean difference vs stable-high group, kPa (95% CI)	Statistic	*P* value
Stable high	7.25 (7.02–7.48)	Reference	—	—
Increasing	7.77 (7.50–8.04)	0.52 (0.10–0.94)	*t* = 2.430	0.016
Persistently low	8.32 (8.07–8.57)	1.07 (0.66–1.48)	*t* = 5.130	<0.001
Overall trajectory-class effect	—	—	*F* = 13.760; *df* = 2, 263	<0.001

The primary analysis used analysis of covariance with follow-up LSM as the dependent variable and adjusted for landmark LSM, the actual follow-up interval, age, sex, diabetes duration, baseline BMI, change in BMI, time-weighted HbA1c, time-weighted triglycerides, time-weighted ALT, time-weighted eGFR, baseline CAP, the two-part vitamin D supplementation variables, and medication exposures. Adjusted marginal means are model-estimated means standardized to the covariate distribution; adjusted mean differences are the differences in marginal means between each trajectory group and the stable-high group. *t* denotes the statistic for a between-group contrast, *F* denotes the statistic for the overall trajectory-class effect, *df* denotes degrees of freedom, and *P* denotes the two-sided test probability. Homogeneity of regression slopes was tested using the trajectory class × landmark LSM interaction. Heteroskedasticity-consistent type 3 (HC3) robust standard errors and 50 pseudo-class draws were used for robustness evaluation. ANCOVA, analysis of covariance; 25(OH)D, 25-hydroxyvitamin D; LSM, liver stiffness measurement; BMI, body mass index; HbA1c, glycated hemoglobin; ALT, alanine aminotransferase; eGFR, estimated glomerular filtration rate; CAP, controlled attenuation parameter; CI, confidence interval.

**Figure 2:**
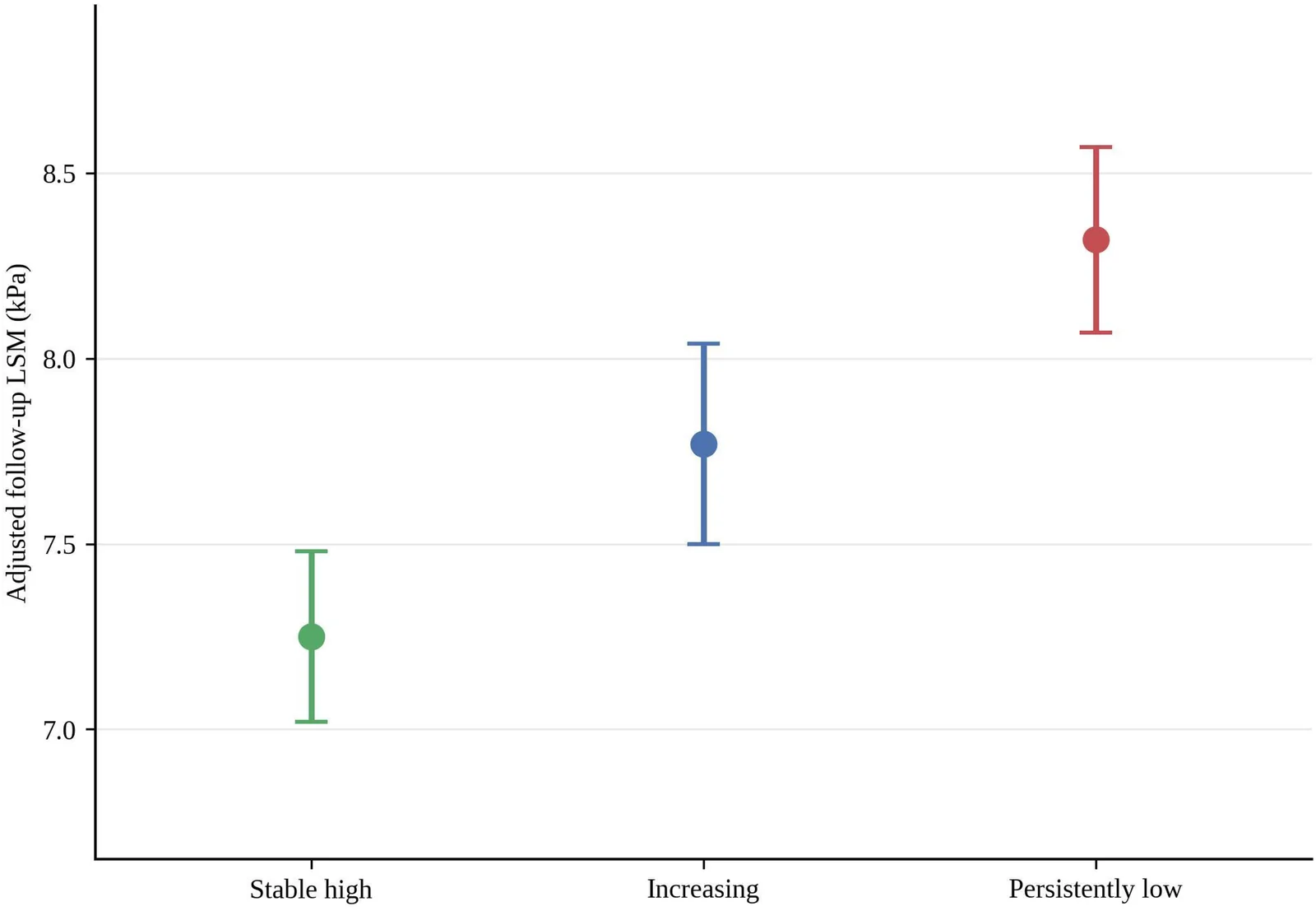
Adjusted follow-up liver stiffness measurement (LSM) across serum 25-hydroxyvitamin D [25(OH)D] trajectory groups. The x-axis shows the stable-high (*n* = 107), increasing (*n* = 84), and persistently low (*n* = 95) trajectories, and the y-axis shows follow-up LSM (kPa) estimated using analysis of covariance (ANCOVA). Points indicate adjusted marginal means, and error bars indicate 95% confidence intervals (CIs). The model adjusted for landmark LSM, the actual follow-up interval, age, sex, diabetes duration, baseline body mass index (BMI), change in BMI, time-weighted glycated hemoglobin (HbA1c), time-weighted triglycerides, time-weighted alanine aminotransferase (ALT), time-weighted estimated glomerular filtration rate (eGFR), baseline controlled attenuation parameter (CAP), the two-part vitamin D supplementation variables, and medication exposures.

### 3.8 Sensitivity analyses

The association for the increasing trajectory was statistically significant in the maximum-posterior-probability hard-classification analysis, the model omitting study-baseline LSM, and the primary adjusted model. Its 95% CI crossed 1 after exclusion of supplemented patients, use of increased LSM risk category or the conservative outcome, restriction to posterior probabilities of at least 0.80, and additional adjustment for landmark LSM. The persistently low trajectory was associated with higher odds in all sensitivity analyses, with a consistent direction of effect (**Table 6**). In the segmented linear mixed-effects model, the adjusted annual changes in LSM from baseline to landmark and from landmark to follow-up were 0.08 kPa/year (95% CI, −0.02 to 0.18; *P* = 0.110) and 1.06 kPa/year (95% CI, 0.90–1.22; *P* < 0.001), respectively; the difference between phase-specific slopes was 0.98 kPa/year (95% CI, 0.80–1.16; *P* < 0.001). After exclusion of the seven patients in the highest 2.5% of absolute landmark-to-follow-up LSM change, the slope difference was 0.86 kPa/year (95% CI, 0.68–1.04; *P* < 0.001) (**Supplementary Table S4**).

**Table 6:** Sensitivity analyses of the association between 25(OH)D trajectories and prespecified LSM increase.

Analysis	Sample size/events	Increasing trajectory OR (95% CI)	Wald *z*; *P* value	Persistently low trajectory OR (95% CI)	Wald *z*; *P* value
Primary adjusted model	286/100	1.98 (1.03–3.82)	2.042; 0.041	3.34 (1.68–6.61)	3.453; <0.001
Maximum-posterior-probability hard classification	286/100	2.04 (1.11–3.75)	2.296; 0.022	3.48 (1.84–6.59)	3.832; <0.001
Excluding vitamin D supplementation	195/72	2.12 (0.94–4.80)	1.807; 0.071	3.43 (1.60–7.34)	3.172; 0.002
Alternative outcome: increase in LSM risk category	286/85	1.67 (0.84–3.31)	1.466; 0.143	2.48 (1.25–4.92)	2.598; 0.009
Conservative LSM increase outcome	286/55	1.71 (0.76–3.84)	1.298; 0.194	3.18 (1.39–7.27)	2.741; 0.006
Posterior classification probability ≥0.80	267/93	1.87 (0.95–3.69)	1.808; 0.071	3.26 (1.67–6.37)	3.460; <0.001
Without adjustment for study-baseline LSM	286/100	2.01 (1.06–3.82)	2.135; 0.033	3.46 (1.80–6.66)	3.719; <0.001
Additional adjustment for landmark LSM	286/100	1.78 (0.91–3.48)	1.685; 0.092	2.91 (1.48–5.71)	3.101; 0.002

The stable-high trajectory was the reference. Sample size/events indicates the number of patients included in the corresponding analysis and the number who met the outcome. Except for the maximum-posterior-probability hard-classification analysis, trajectory effects were based on 50 pseudo-class draws, with within-draw and between-draw variances combined using Rubin’s rules. The conservative LSM increase outcome was analyzed using Firth penalized maximum-likelihood logistic regression; the remaining binary sensitivity analyses used standard logistic regression. OR denotes odds ratio, 95% CI denotes confidence interval, Wald *z* is the regression coefficient divided by its pooled standard error, and *P* is the two-sided Wald-test probability based on a large-sample normal approximation. 25(OH)D, 25-hydroxyvitamin D; LSM, liver stiffness measurement; OR, odds ratio; CI, confidence interval.

### 3.9 Exploratory interaction analyses

Exploratory interaction analyses based on maximum-posterior-probability hard classification showed that, among men and women and among patients with and without obesity, the conditional effects for the persistently low trajectory were consistently greater than those for the stable-high trajectory. After Holm adjustment, the interactions of trajectory class with sex and obesity status were not statistically significant (both *P* > 0.05) (**Figure 3**; **Supplementary Table S5**).

**Figure 3:**
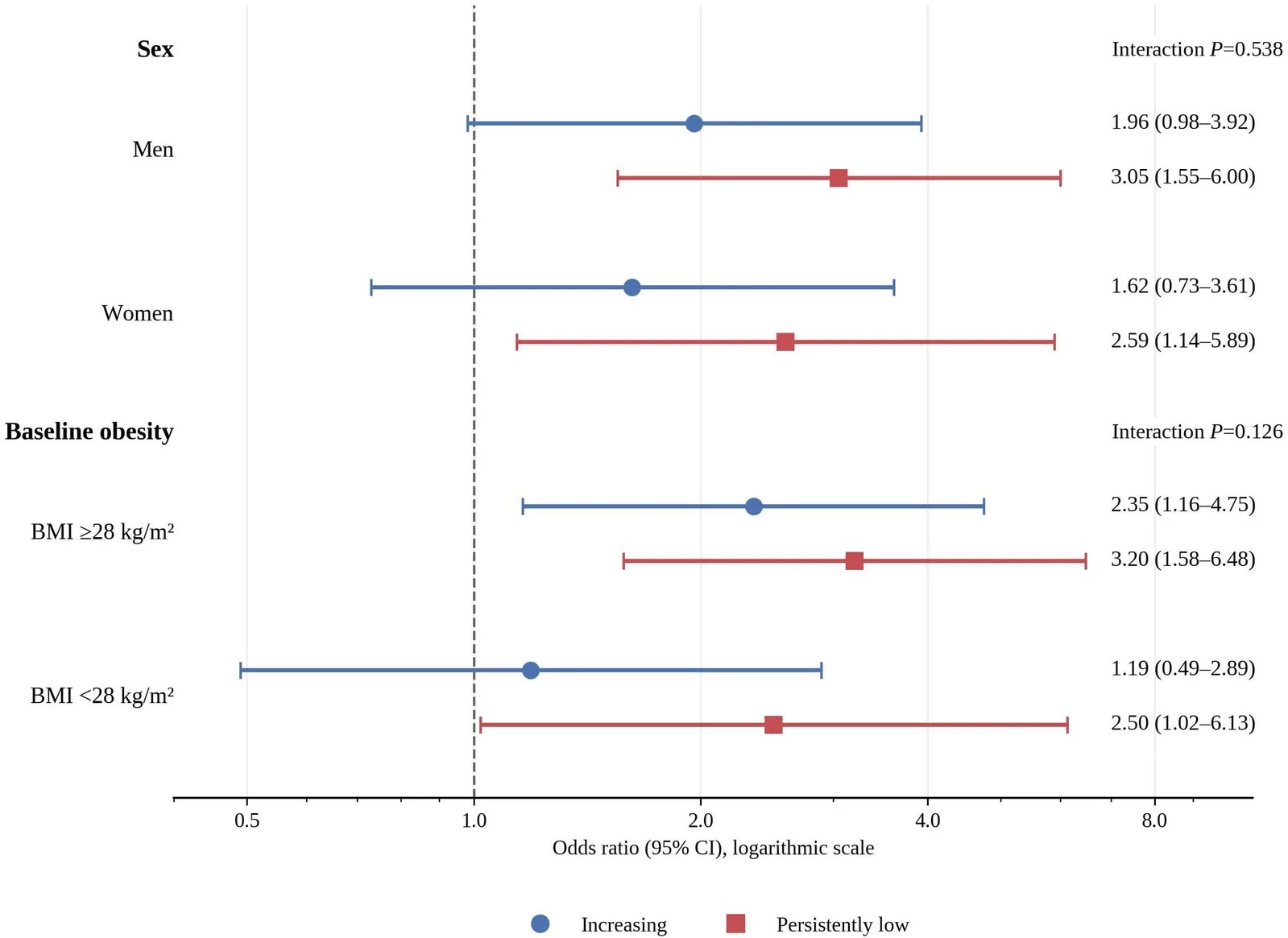
Exploratory interaction analyses of the association between serum 25-hydroxyvitamin D [25(OH)D] trajectories and prespecified liver stiffness measurement (LSM) increase. The x-axis of the forest plot uses a logarithmic scale. Circles and squares indicate conditional odds ratios (ORs) for the increasing and persistently low trajectories, respectively, relative to the stable-high trajectory; horizontal lines indicate 95% confidence intervals (CIs), and the vertical dashed line indicates OR = 1. The sample size/events in the four subgroups of men, women, baseline body mass index (BMI) ≥28 kg/m², and BMI <28 kg/m² were 164/56, 122/44, 164/59, and 122/41, respectively. Holm-adjusted *P* values for the interactions with sex and obesity status were 0.538 and 0.126, respectively. This exploratory analysis used maximum-posterior-probability hard classification.

## 4 Discussion

Among patients with early-onset type 2 diabetes and MASLD who completed standardized repeated assessments, this study identified three 25(OH)D trajectories: persistently low, increasing, and stable high. After within-draw and between-draw variances were combined using Rubin’s rules, the persistently low trajectory showed the most consistent associations with prespecified post-landmark LSM increase and higher follow-up LSM. The association for the increasing trajectory was weaker than that for the persistently low trajectory in the primary model, and its 95% CI included 1 in analyses using the extended adjustment, conservative outcome, increased LSM risk category, and additional adjustment for landmark LSM. Existing clinical evidence indicates that lower 25(OH)D levels coexist with fatty liver and higher fibrosis indices in patients with type 2 diabetes (21). Synthesized evidence in patients with MASLD and type 2 diabetes also suggests generally lower vitamin D levels, although effect sizes vary across populations and definitions (22).

The increasing trajectory had both the highest prevalence of vitamin D supplementation and the greatest reduction in BMI. Within-trajectory stratification showed that 25(OH)D increased over time in both subgroups, but the increase was greater among supplemented patients, suggesting that this trajectory may simultaneously reflect vitamin D supplementation, weight change, and unmeasured concurrent health behaviors. Existing evidence suggests that vitamin D supplementation may modify some fibrosis-related molecular markers but has not shown consistent improvement in clinical liver-stiffness outcomes (23). Synthesized evidence from randomized trials in chronic liver disease mainly indicates improvements in selected biochemical and metabolic indices, whereas evidence regarding liver stiffness, hepatic steatosis, and clinical liver outcomes remains limited (24). Available genetic evidence has likewise not supported a clear causal effect of circulating 25(OH)D on steatotic liver disease (25).

Circulating 25(OH)D may also reflect adiposity, outdoor activity, sunlight exposure, and dietary intake. Lower 25(OH)D has been associated with a higher probability of steatotic liver disease in people with metabolic dysfunction, although adiposity and other metabolic abnormalities may jointly influence this association (26). Diet, objectively measured physical activity, duration of sunlight exposure, and actual adherence to supplementation were not adequately measured; residual confounding and reverse causation may therefore continue to affect the trajectory associations. Biological links between vitamin D and hepatic inflammation, oxidative stress, and fibrogenic pathways support an interpretation based on association but are insufficient by themselves to establish causality (27).

In the extended model, time-weighted HbA1c, baseline CAP, and study-baseline LSM remained statistically associated with the outcome; the 95% CI for baseline BMI included 1, and *P* = 0.060. Diabetes-related insulin resistance, lipotoxicity, mitochondrial dysfunction, and proinflammatory signaling may jointly increase susceptibility to hepatic fibrosis (28). Weight change is associated with the risk of liver fibrosis in patients with type 2 diabetes, indicating that longitudinal weight control is an important contextual factor when interpreting the increasing trajectory (29). The intestinal barrier, microbial metabolites, and low-grade inflammation may link dysregulated glucose metabolism to hepatic lipid accumulation (30). Metabolic subtypes of type 2 diabetes differ in obesity, insulin resistance, and insulin deficiency, and their hepatic susceptibility may likewise differ (31).

The 1-year incidence of the prespecified LSM increase was 35.0%, reflecting a selected population that entered a standardized diabetes–liver follow-up pathway and completed serial transient elastography. The segmented model showed a steeper LSM slope from the landmark to follow-up than from baseline to the landmark, and the difference remained after excluding patients in the highest 2.5% of absolute change during the later phase, indicating that the temporal pattern was not entirely driven by a small number of extreme values. However, the retrospective design cannot separate the relative contributions of true short-term changes in liver stiffness, informative follow-up, transient-elastography measurement variability, and regression to the mean. The conservative outcome required both a relative increase of at least 30% and an absolute increase of at least 3.0 kPa; the overall event rate decreased to 19.2%, while the association with the persistently low trajectory remained. Noninvasive serum scores and imaging markers are reproducible and minimally invasive, but their accuracy depends on the disease spectrum and threshold selection (32). The performance of noninvasive tests for identifying advanced fibrosis varies (33). The accuracy of commonly used noninvasive scores is not constant across populations, and changes in a single measure should be interpreted in the context of initial liver stiffness, metabolic status, and quality control (34).

The prevalence of advanced fibrosis and occult cirrhosis is high among people with type 2 diabetes, supporting proactive screening and sequential assessment within clinical pathways (35). Long-term histopathologic evidence indicates that diabetes is associated with faster fibrosis progression, but this process and 1-year changes on transient elastography represent different outcome scales (36). The analytical objective of this study was to evaluate the longitudinal association of 25(OH)D trajectories with LSM increase; discrimination, calibration, and clinical net benefit are domains of prediction-model evaluation. The incremental predictive value of 25(OH)D trajectories beyond existing noninvasive scores, BMI, HbA1c, CAP, and LSM requires validation using prespecified models in independent prospective samples.

This study has several limitations. First, the retrospective design and standardized follow-up pathway may have introduced selection bias, and the results are primarily applicable to patients with similar repeated-assessment conditions. Second, diet, objectively measured physical activity, sunlight exposure, actual adherence to supplementation, and genetic susceptibility were not adequately measured, and residual confounding remains possible. Third, the trajectories were constructed from a limited number of electrochemiluminescence measurements. Additional tests arose from clinical management; although their frequency did not differ significantly according to baseline vitamin D deficiency, classification bias from informative testing and timing error cannot be fully excluded. Fourth, the outcomes reflect changes in LSM measured by transient elastography without histologic or magnetic resonance elastography validation, and measurement variability and regression to the mean in the lower LSM range may still have influenced the estimates. Fifth, follow-up was relatively short and did not permit evaluation of long-term outcomes such as cirrhosis, liver-related events, or death.

## 5 Conclusions

Distinct longitudinal serum 25(OH)D trajectories were present among patients with early-onset type 2 diabetes and MASLD. The persistently low trajectory showed the most consistent associations with prespecified post-landmark LSM increase and higher follow-up LSM, whereas associations for the increasing trajectory varied according to supplementation, weight change, model adjustment, and outcome definition. The findings support interpretation as longitudinal associations but do not support definitive inferences regarding causal effects, effects of vitamin D supplementation, or individual-level predictive performance.

## Data Availability

The raw data supporting the conclusions of this article will be made available by the authors, without undue reservation.
