# Supplementary material for: Association of longitudinal serum 25-hydroxyvitamin D trajectories with subsequent liver stiffness increase in patients with early-onset type 2 diabetes and metabolic dysfunction-associated steatotic liver disease
Source: Front Endocrinol (Lausanne). 2026 Oct 28;17:1987830. doi: 10.3389/fendo.2026.1987830 (PMC13752080; doi:10.3389/fendo.2026.1987830)
Supplement: Supplementary file 1 [file Table1.docx]

**Supplementary Table S1. Fit Statistics for Candidate Longitudinal 25(OH)D Trajectory Models**

| Number of classes | Time form | Number of parameters | Log-likelihood | AIC | BIC | Entropy | Minimum class proportion (%) | Lowest mean posterior probability |
| --- | --- | --- | --- | --- | --- | --- | --- | --- |
| 2 | Linear | 9 | −2540.6 | 5099.2 | 5132.1 | 0.74 | 38.5 | 0.87 |
| 2 | Quadratic | 11 | −2518.4 | 5058.8 | 5099.0 | 0.78 | 38.1 | 0.89 |
| 3 | Linear | 13 | −2490.2 | 5006.4 | 5053.9 | 0.80 | 27.6 | 0.82 |
| 3 | Quadratic | 16 | −2461.7 | 4955.4 | 5013.9 | 0.86 | 29.4 | 0.86 |
| 4 | Linear | 17 | −2478.3 | 4990.6 | 5052.8 | 0.79 | 5.6 | 0.75 |
| 4 | Quadratic | 21 | −2448.2 | 4938.4 | 5015.2 | 0.80 | 4.2 | 0.72 |
| 5 | Linear | 21 | −2464.1 | 4970.2 | 5047.0 | 0.75 | 3.5 | 0.69 |
| 5 | Quadratic | 26 | −2439.8 | 4931.6 | 5026.7 | 0.76 | 2.8 | 0.68 |

**Note:** All models included a subject-specific random intercept and sine and cosine terms for month of blood collection. Number of parameters denotes the total number of freely estimated model parameters; a higher log-likelihood indicates better fit; AIC and BIC are information criteria that penalize model complexity, with lower values indicating better relative fit; entropy ranges from 0 to 1, with values closer to 1 indicating better class separation; minimum class proportion is the percentage of the total sample in the smallest class; and lowest mean posterior probability is the smallest mean posterior classification probability across classes. These indices were used for model selection rather than hypothesis testing; therefore, P values are not reported. 25(OH)D, 25-hydroxyvitamin D; AIC, Akaike information criterion; BIC, Bayesian information criterion.
