# Supplementary material for: Association of longitudinal serum 25-hydroxyvitamin D trajectories with subsequent liver stiffness increase in patients with early-onset type 2 diabetes and metabolic dysfunction-associated steatotic liver disease
Source: Front Endocrinol (Lausanne). 2026 Oct 28;17:1987830. doi: 10.3389/fendo.2026.1987830 (PMC13752080; doi:10.3389/fendo.2026.1987830)
Supplement: Supplementary file 2 [file Table2.docx]

**Supplementary Table S2. Robustness Analysis of the Random-Slope Latent Class Model**

| Model | Number of parameters | Log-likelihood | AIC | BIC | Entropy | Minimum class proportion | Lowest mean posterior probability | Concordance with primary model | Adjusted Rand index |
| --- | --- | --- | --- | --- | --- | --- | --- | --- | --- |
| Three-class quadratic time: random intercept | 16 | −2461.7 | 4955.4 | 5013.9 | 0.86 | 29.4% | 0.86 | — | — |
| Three-class quadratic time: random intercept + random slope | 20 | −2452.8 | 4945.6 | 5018.7 | 0.84 | 28.7% | 0.84 | 262/286 (91.6%) | 0.81 |

**Note:** The random-slope model used an unstructured intercept–slope covariance and allowed class-specific random-effect scales. Number of parameters, log-likelihood, AIC, BIC, entropy, minimum class proportion, and lowest mean posterior probability are defined as in Supplementary Table S1. Concordance with the primary model is the proportion of patients assigned to the same trajectory class by both models. The adjusted Rand index ranges from −1 to 1, with values closer to 1 indicating greater classification agreement. This table evaluates model robustness and does not present hypothesis tests. AIC, Akaike information criterion; BIC, Bayesian information criterion.
