# Supplementary material for: Association of longitudinal serum 25-hydroxyvitamin D trajectories with subsequent liver stiffness increase in patients with early-onset type 2 diabetes and metabolic dysfunction-associated steatotic liver disease
Source: Front Endocrinol (Lausanne). 2026 Oct 28;17:1987830. doi: 10.3389/fendo.2026.1987830 (PMC13752080; doi:10.3389/fendo.2026.1987830)
Supplement: Supplementary file 3 [file Table3.docx]

**Supplementary Table S3. Changes in 25(OH)D Within the Increasing Trajectory, Stratified by Vitamin D Supplementation Status**

| Time point or measure | Vitamin D supplementation (n = 50) | No vitamin D supplementation (n = 34) | Between-group comparison |
| --- | --- | --- | --- |
| Baseline 25(OH)D, ng/mL | 16.3 ± 2.8 | 17.0 ± 3.0 | Holm-adjusted P = 0.285 |
| 6-month 25(OH)D, ng/mL | 20.1 ± 2.6 | 18.2 ± 2.7 | Holm-adjusted P = 0.004 |
| 12-month 25(OH)D, ng/mL | 24.8 ± 2.0 | 21.6 ± 2.0 | Holm-adjusted P<0.001 |
| Change from baseline to 12 months, ng/mL | 8.5 ± 2.7 | 4.6 ± 2.8 | Mean difference, 3.9 (95% CI, 2.68–5.12); P<0.001 |

**Note:** Data are presented as mean ± standard deviation; the mean indicates the average within-group level, and the standard deviation indicates dispersion of individual observations around the mean. Between-group comparisons at baseline, 6 months, and 12 months used Welch t tests, with Holm adjustment of the P values for the three time points; change from baseline to 12 months was compared using a Welch t test. The difference is the supplemented-group mean minus the non-supplemented-group mean, 95% CI denotes the confidence interval for the difference, and P values are two-sided test probabilities. This stratification was descriptive. 25(OH)D, 25-hydroxyvitamin D; CI, confidence interval.
