# Supplementary material for: Association of longitudinal serum 25-hydroxyvitamin D trajectories with subsequent liver stiffness increase in patients with early-onset type 2 diabetes and metabolic dysfunction-associated steatotic liver disease
Source: Front Endocrinol (Lausanne). 2026 Oct 28;17:1987830. doi: 10.3389/fendo.2026.1987830 (PMC13752080; doi:10.3389/fendo.2026.1987830)
Supplement: Supplementary file 4 [file Table4.docx]

**Supplementary Table S4. Segmented Slopes Across the Two LSM Observation Phases and Extreme-Value Sensitivity Analysis**

| Analysis | Sample size | Estimate (kPa/year) | SE | 95% CI | Wald z; P value |
| --- | --- | --- | --- | --- | --- |
| Baseline-to-landmark phase | 286 | 0.08 | 0.05 | −0.02–0.18 | 1.600; 0.110 |
| Landmark-to-follow-up phase | 286 | 1.06 | 0.08 | 0.90–1.22 | 13.250; <0.001 |
| Difference between later- and earlier-phase slopes | 286 | 0.98 | 0.09 | 0.80–1.16 | 10.889; <0.001 |
| Slope difference after excluding the highest 2.5% of absolute change in the later phase | 279 | 0.86 | 0.09 | 0.68–1.04 | 9.556; <0.001 |

**Note:** A segmented linear mixed-effects model with a knot at the landmark was used. Time was calculated in years from the actual examination dates. The model included a subject-specific random intercept and adjusted for age, sex, diabetes duration, baseline BMI, change in BMI, time-weighted HbA1c, and baseline CAP. The slope difference was calculated as the landmark-to-follow-up slope minus the baseline-to-landmark slope. The extreme-value sensitivity analysis excluded the seven patients in the highest 2.5% of absolute landmark-to-follow-up LSM change. SE, standard error; CI, confidence interval; LSM, liver stiffness measurement; BMI, body mass index; HbA1c, glycated hemoglobin; CAP, controlled attenuation parameter.
