# Supplementary material for: Association of longitudinal serum 25-hydroxyvitamin D trajectories with subsequent liver stiffness increase in patients with early-onset type 2 diabetes and metabolic dysfunction-associated steatotic liver disease
Source: Front Endocrinol (Lausanne). 2026 Oct 28;17:1987830. doi: 10.3389/fendo.2026.1987830 (PMC13752080; doi:10.3389/fendo.2026.1987830)
Supplement: Supplementary file 5 [file Table5.docx]

**Supplementary Table S5. Exploratory Interaction Analyses by Sex and Obesity Status**

| Stratification factor | Subgroup | Sample size/events | Increasing trajectory OR (95% CI); P value | Persistently low trajectory OR (95% CI); P value | Interaction test |
| --- | --- | --- | --- | --- | --- |
| Sex | Men | 164/56 | 1.96 (0.98–3.92); P = 0.057 | 3.05 (1.55–6.00); P = 0.001 | χ² = 1.240; Holm-adjusted P = 0.538 |
| Sex | Women | 122/44 | 1.62 (0.73–3.61); P = 0.237 | 2.59 (1.14–5.89); P = 0.023 | As above |
| Obesity status | BMI≥28 kg/m² | 164/59 | 2.35 (1.16–4.75); P = 0.018 | 3.20 (1.58–6.48); P = 0.001 | χ² = 5.540; Holm-adjusted P = 0.126 |
| Obesity status | BMI<28 kg/m² | 122/41 | 1.19 (0.49–2.89); P = 0.701 | 2.50 (1.02–6.13); P = 0.045 | As above |

**Note:** This exploratory analysis used maximum-posterior-probability hard classification. Sample size/events indicates the number of patients in each subgroup and the number who met the prespecified LSM increase outcome. Conditional effects were obtained from full-sample Firth penalized logistic regression models containing trajectory class, the stratification factor, and their interaction. The sex model adjusted for all covariates in the primary model except sex; the obesity model replaced continuous BMI with an obesity indicator. OR denotes conditional odds ratio, 95% CI denotes confidence interval, χ² is the joint Wald statistic for the interaction, and P values are two-sided test probabilities; the P values for the two interaction tests were adjusted using the Holm method. 25(OH)D, 25-hydroxyvitamin D; BMI, body mass index; LSM, liver stiffness measurement; OR, odds ratio; CI, confidence interval.
